# Bortezomib Congeners Induce Apoptosis of Hepatocellular Carcinoma via CIP2A Inhibition

**DOI:** 10.3390/molecules181215398

**Published:** 2013-12-11

**Authors:** Duen-Ren Hou, Ann-Chi Huang, Chung-Wai Shiau, Chun-Yi Wang, Hui-Chuan Yu, Kuen-Feng Chen

**Affiliations:** 1 Department of Chemistry, National Central University, Taoyuan 32001, Taiwan; E-Mail: drhou@ncu.edu.tw (D.-R.H.); achuang@cc.ncu.edu.tw (A.-C.H.); cywang@cc.ncu.edu.tw (C.-Y.W.); 2 Institute of Biopharmaceutical Sciences, National Yang-Ming University, Taipei 11221, Taiwan; E-Mail: cwshiau@ym.edu.tw; 3 Department of Medical Research, National Taiwan University Hospital, Taipei 10048, Taiwan; E-Mail: gane-mai@yahoo.com.tw; 4 National Center of Excellence for Clinical Trial and Research, National Taiwan University Hospital, Taipei 10002, Taiwan

**Keywords:** CIP2A, bortezomib, apoptosis

## Abstract

CIP2A is an oncoprotein that upregulates p-Akt and promotes cancer cell proliferation and survival. The proteasome inhibitor bortezomib has been shown to reduce CIP2A and lead to cell apoptosis. Here; we modified the functional group of bortezomib to generate a series of novel compounds and conducted a structure–activity relationship (SAR) study. The results showed that compound **1** was able to repress CIP2A expression and cell apoptosis in the same manner as bortezomib, but with less potency in inhibition of proteasome activity. This finding provides a new direction for the design of CIP2A inhibitors.

## 1. Introduction

Cancerous inhibitor of protein phosphatase 2A (CIP2A) is a human oncoprotein which is overexpressed in many cancers, including hepatocellular carcinoma (HCC), lung, colon, prostate, ovarian, cervical, breast, head and neck cancers and leukemia [1]. Overexpression of CIP2A in transformed human cells promotes anchorage-independent cell growth and *in vivo* tumor formation [2]. A mechanistic study showed that CIP2A inhibits protein phosphatase activity which reduces PP2A-mediated dephosphorylation of c-myc or Akt. This inhibition prevents c-myc degradation, maintains Akt activity and further promotes cell progression [3,4,5,6]. 

A natural product, rabdocoetsin B, has been reported to inhibit CIP2A level and reduce cancer cell proliferation [7]. Although rabdocoetsin B shows CIP2A inhibition activity, the pure compound is not easy to obtain and purify. In addition, designing a synthetic route for rabdocoetsin B is complicated by the chiral centers in its structure. Moreover, this natural product may induce off-target effects and activation or inactivation of other enzyme activity at high concentrations.

**Figure 1:**
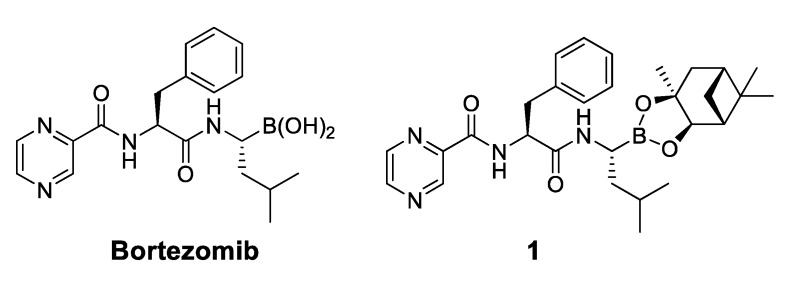
Chemical structures of bortezomib and compound **1**.

Botezomib (Velcade^®^, Millennium Pharmaceuticals, Inc., Cambridge, MA, USA, Figure 1) is a proteasome inhibitor used clinically for relapsed multiple myeloma and mantle cell lymphoma [8]. Studies have shown that bortezomib is able to reduce the phosphorylation level of Akt in HCC [4]. Further study showed that bortezomib reduced p-Akt through activating PP2A phosphatase activity and downregulating CIP2A expression in HCC cells [9,10]. To date, no correlation has been found between proteasome inhibition and inhibition of the CIP2A/PP2A/Akt pathway by bortezomib. In addition, there are no reports showing the relationship between CIP2A expression and proteasome activity induced by bortezomib derivatives that inhibit CIP2A expression. 

In light of the ability of bortezomib to reduce CIP2A level, here we synthesized some novel bortezomib derivatives that retain the ability to reduce cell survival, but with less reduction of proteasome activity than bortezomib. We provide data showing that these novel bortezomib derivatives mediate apoptosis correlated with downregulation of CIP2A. 

## 2. Results and Discussion

### 2.1. Chemistry

**Scheme 1:**
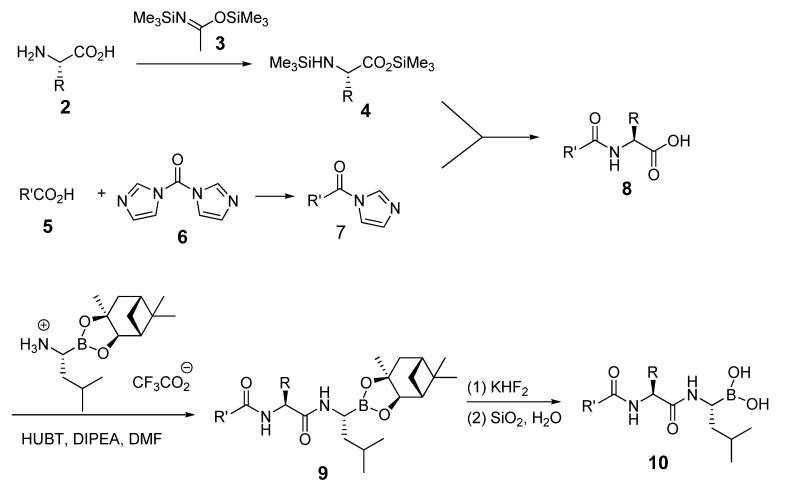
Synthesis of bortezomib derivatives.

Bortezomib is a modified dipeptide that can be written as Pyz-Phe-boroLeu, where the N-terminus is capped by pyrazinoic acid and the carboxylic acid of leucine is replaced with a boronic acid moiety. The boronic acid plays a crucial role in inhibiting 20S activity. For example, the boron atom acts as an electrophile to covalently interact with the nucleophilic oxygen of Thr10. In addition, Thr1 of the proteasome generates a hydrogen bridge with the boronate hydroxyl group further stabilizing the whole complex. To elucidate the relationship between proteasome activity and downregulation of CIP2A, we used a chemical approach to reduce the interaction between the boronic acid of bortezomib and Thr1 of the proteasome by adding a bulky group to the boronic acid. The proteasome activity of the resulting bortezomib derivative (**1**, Figure 1) was tested by ELISA. To explore the structure-activity relationship of the downregulation of CIP2A, we replaced the boronic acid of bortezomib with various functional groups yielding compounds **11**–**14**. Moreover, we replaced the pyrazinoic ring with benzene and methyl groups and used it as a platform to carry out structural modifications, which generated compounds **15**–**20**. These bortezomib derivatives were synthesized according to a general procedure which is described in Scheme 1. The inhibition of CIP2A and the proteasome activity of these compounds were tested by ELISA and western blot.

#### The Synthetic Route to Bortezomib Derivatives

The original synthesis of bortezomib started with the pinanediol ester of leucine boronic acid; however, we started with a carboxylic acid reaction with 1,1′-carbonyldiimidazole that was further coupled with *N*,*O*-Bis(trimethylsilyl)acetamide. The intermediate product was incorporated with a boronic ester to generate compound **4**. To investigate the CIP2A inhibition ability of boronic acid and boronic ester, we hydrolyzed the boronic esters with the sequence of potassium hydrogen difluoride and silica gel to obtain boronic acid compounds in high yield. 

### 2.2. Biological Evaluations

#### 2.2.1. Development of a Bortezomib Derivative with a Bulky Pinanediol Group

**Figure 2:**
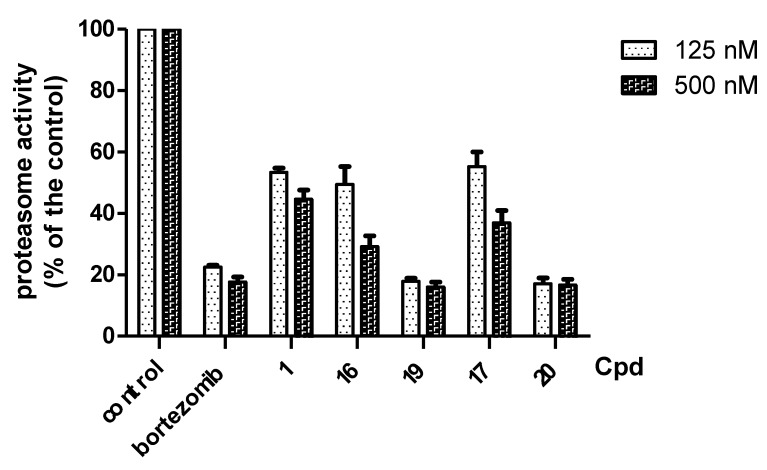
Effects of bortezomib and its derivatives on proteasome inhibition. Sk-hep1 cells were exposed to 100 nM bortezomib and compounds **1**, **16**, **17**, **19**, and **20** for 5 h before measurement of proteasome activity. *Columns*, mean (*n = 3*); *bars*, SD.

We synthesized a bortezomib derivative **1** with a bulky pinanediol group connected to the boron atom which reduces its hydrogen acceptor and donor ability. Theoretically, compound **1** should have less binding affinity than bortezomib to the proteasome Thr 10 and Thr1 residues and less cell toxicity. We then explored the ability of bortezomib and compound **1** to inhibit proteasome activity in Sk-Hep1 cells. Bortezomib was able to inhibit 75% of the proteasome activity of untreated Sk-Hep1 cells at 100 nM (Figure 2). In contrast, compound **1**-treated cells showed only 40% inhibitory activity as compared to the vehicle control.

#### 2.2.2. Structure-Activity Relationship between Boronic Acid and the Pyrazinoic Ring, and Cell Growth Inhibition

The boronic acid functional group of bortezomib was replaced with ester and hydroxylmethylene groups, generating compounds **11** and **12** (Table 1). None of these derivatives with hydrogen donor and acceptor groups showed greater cell toxicity than bortozomib and compound **1**. The isobutyl group is also an important factor in cell growth inhibition. For example, compounds **13** and **14** which have no isobutyl group have no growth inhibition effect. Next, we replaced the pyrazinoic ring with a benzene ring and a methyl group to generate compounds **16** and **17** (Table 2). The benzene ring showed the same growth inhibitory effect as the pyrazinoic ring whereas the methyl group showed less cytotoxicity. We also generated compound **15** by removing the benzyl group at the R2 position, thus dramatically reducing the cytotoxicity. We further hydrolyzed the pinanediol group of compounds **15**, **16**, and **17** at the boron position resulting in compounds **18**, **19** and **20**, respectively (Table 3). Compounds **18** and **19** showed similar effect to compounds **15**–**17** in inducing cell death.

**Table 1:** Chemical structure of synthetized compounds and IC_50_ of growth inhibition in Sk-hep-1 cells. 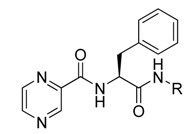

Cpd	R	IC 50 (nM) in Sk-hep1 Cells
**Bortezomib**	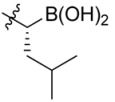	105
**1**	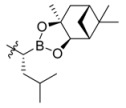	55
**11**	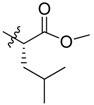	>1000
**12**	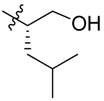	>1000
**13**	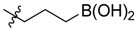	>1000
**14**	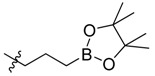	>1000

**Table 2:** Chemical structure of synthetized compounds and IC_50_ of growth inhibition in Sk-hep-1 cells. 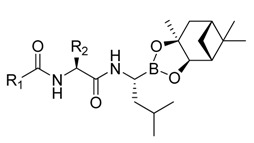

Cpd	R_1_	R_2_	IC 50 (nM) in Sk-hep1 Cells
**15**	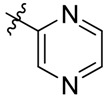	H	>1000
**16**	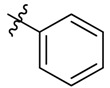	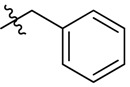	58.7
**17**	CH_3_	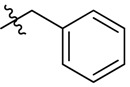	432.6

**Table 3:** Chemical structure of synthetized compounds and IC_50_ of growth inhibition in Sk-hep-1 cells. 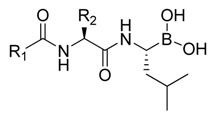

Cpd	R_1_	R_2_	IC 50 (nM) in Sk-hep1 Cells
**18**	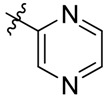	H	>1000
**19**	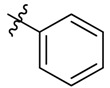	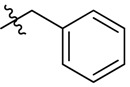	177.8
**20**	CH_3_	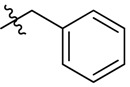	314.6

#### 2.2.3. Target Validation of Bortezomib Derivatives

**Figure 3:**
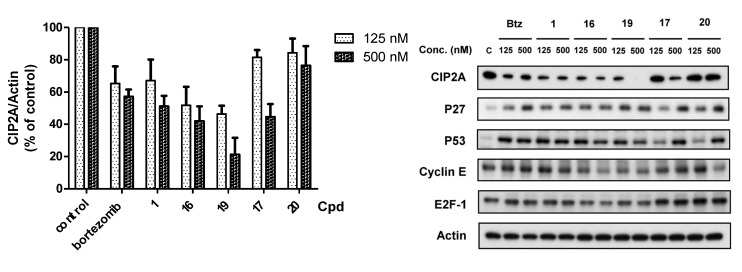
Effects of bortezomib and derivatives on protein levels of CIP2A in Sk-hep1 cells. Cells were exposed to bortezomib and derivatives at the indicated doses for 24 h. Cell lysates were assayed by Western blot. Immunoblots were scanned by a UVP BioSpectrum AC image system and quantitated using VisionWork LS software to determine the ratio of the level of CIP2A to actin. *Columns*, mean (*n* = 3); *bars*, SD.

The bortezomib derivative with a boron-pinanediol component showed the same potency against cancer cell growth as bortezomib. However, some of the compounds showed less potent proteasome inhibition activity. Therefore, we hypothesized that the bortezomib derivatives may enhance cell death by downregulatation of CIP2A. The bortezomib derivatives were tested for inhibition of CIP2A expression by western blot. As shown in Figure 3, boron-pinanediol (compounds **1** and **16**) and boronic acid compounds (bortezomib and compound **19**) showed a significant repression of CIP2A expression at 125 nM. Compounds **17** and **20** were able to reduce CIP2A at 500 nM. The result indicated that CIP2A plays an important role in regulating cell survival. At the same time, we also examined proteasome regulation proteins including P27, P53, cyclin E and E2F-1 with drug treatment (Figure 3). 

**Figure 4:**
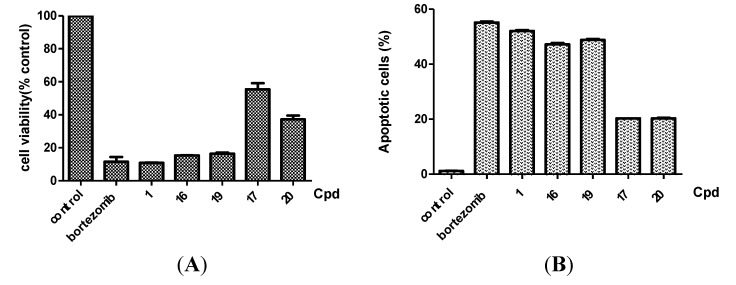
(**A**) Effects of bortezomib and derivatives on cell viability in a Sk-hep1 cell line. Sk-hep1 cells were exposed to bortezomib and derivatives at the indicated concentrations in DMEM with 5% FBS in 96-well plates for 48 h and cell viability was assessed by MTT assay. *Columns*, mean (*n* = 3); *bars*, SD; (**B**) Bortezomib and derivatives induce apoptosis in Sk-hep1 cells. Analysis of apoptotic cells of Sk-hep1 cells was conducted by flow cytometry (sub-G1) after cells were exposed to 500 nM bortezomib and derivatives for 24 h. *Points*, mean; *bars*, S.D. (*n* = 3).

**Figure 5:**
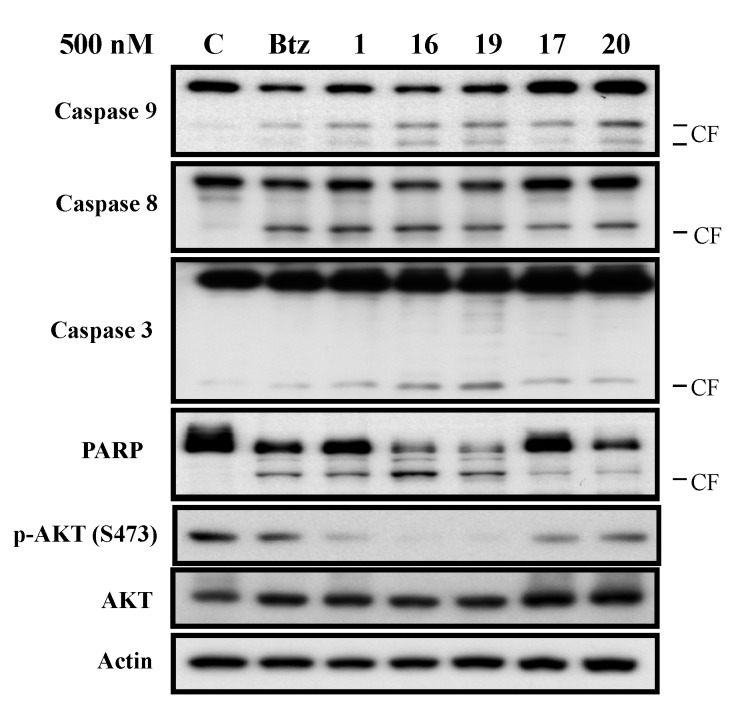
Western blot analysis of Akt (Akt1), p-Akt (Ser473), caspases and PARP levels. Sk-hep1 cells were exposed to bortezomib and derivatives at 500 nM in DMEM with 5% FBS for 24 h. Cell lysates were prepared for Akt (Akt1), p-Akt (Ser473), caspase-8, caspase-9, caspase-3, and PARP. CF, cleaved form (activated form).

**Figure 6:**
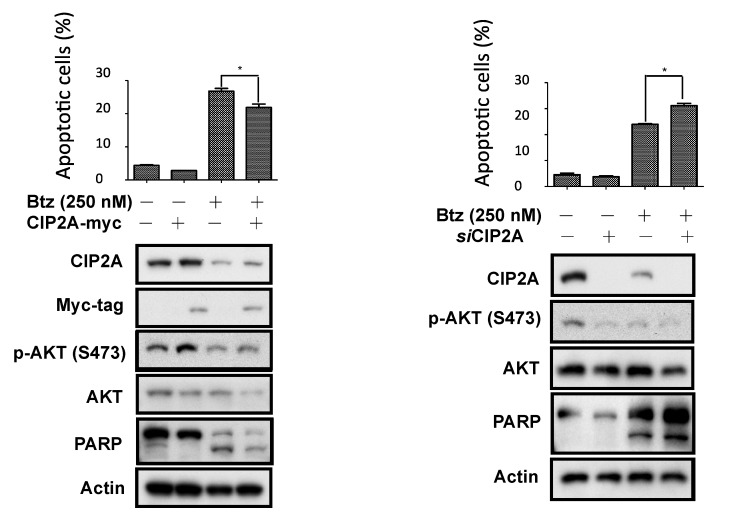
CIP2A mediated brotezomib-induced apoptosis through p-Akt regulation. Cell were transfected with CIP2A siRNA (*right*) and ectopic expression of CIP2A (*left*), then treated with brotezomib (250 nM) for 24 h. cells were analysis by western blot and flow cytometry. *Points*, mean; *bars*, S.D.; * Difference is statistically significant (*p* < 0.05); *n* = 3.

The cell survival was not correlated with the expression of these proteins, suggesting that these proteins were not the major factors in cell survival. We also applied flow cytometry assays to confirm that the decreased level of CIP2A induced by these derivatives correlated with cell toxicity in Sk-hep1 cells (Figure 4). In other words, these derivatives induced cell death in part through inhibition of CIP2A. Moreover, these compounds were tested in Huh7 and Hep3B with MTT assay and the trend of potency in Huh7 and Hep3B is similar to Sh-hep-1 in IC50 (Table 4). In addition, we also tested the downstream signaling pathway. Expression levels of p-Akt, a downstream target of CIP2A, were assessed in bortezomib, and compounds **1**, **16**, **17**, **19**, and **20** at 500 nM. As shown in Figure 5, compounds **1**, **16**, and **19** with CIP2A inhibitory activity, were able to reduce p-Akt levels, and compounds **17**, and **20** had moderate effects on either protein. The activation of caspases and the cleavage of PARP were examined for these agents with western blotting assays. We further explored whether CIP2A plays an important role in regulating cell survival. Genetic overexpression of CIP2A showed a rescue effect on bortezomib-induced cell toxicity. Knockdown of CIP2A increase cell toxicity with the treatment of bortezomib, indicating that CIP2A is one of the major targets of bortezomib (Figure 6). 

**Table 4:** IC_50_ of growth inhibition in Sk-hep-1, Huh-7, and Hep3B cells with bortezomib, **1**, **16**, **17**, **19**, and **20**.

	Cpd	Bortezomib	1	16	19	17	20
Cells MTT IC_50_ (nM)							
Sk-hep-1		105.0	54.9	58.7	177.8	432.6	314.5
Huh 7		273.5	489.4	118.3	158.5	416.5	448.2
Hep 3B		20.7	12.8	5.8	34.3	93.0	31.1

In summary, these data suggest that boron-pinanediol derivative induced-biological activity was due to the inhibition of CIP2A and triggered the apoptotic signals.

### 2.3. Discussion

Recent data suggest that CIP2A is an oncoprotein which activates p-Akt and its downstream cell survival and proliferation signals. This study confirms that bortezomib and its derivatives induce cell death through CIP2A inhibition. These findings have several implications. First, the results confirm that cell death induced by bortezomib and its derivatives does not totally depend on proteasome inhibition. Although bortezomib has been approved by the FDA as a proteasome inhibitor in multiple myeloma and mantle cell lymphoma, its effect on solid tumors was not significant. Our findings suggest that CIP2A is involved in this event. Second, our data suggest that CIP2A is a potential drug target for anticancer agents as the drug sensitivity induced by bortezomib and its derivatives correlated with CIP2A expression. Third, we also found that downregulation of CIP2A by bortezomib and its derivatives results in downregulation of p-Akt, suggesting that CIP2A also influences the interaction between PP2A and its p-Akt substrate. Since CIP2A is also over expressed in other cancers, we plan to explore the CIP2A ablative effect of these compounds before the preclinical study.

## 3. Experimental

### 3.1. Materials

Proton nuclear magnetic resonance (^1^H-NMR) spectra were recorded on a Bruker DPX300 (300 MHz) instrument. Chemical shifts are reported in ppm. Peak multiplicities are expressed as follows: s, singlet; d, doublet; t, triplet; q, quartet; dd, doublet of doublet; ddd, doublet of doublet of doublets; dt, doublet of triplet; brs, broad singlet; m, multiplet. Coupling constants (J values) are given in hertz (Hz). The reaction progress was determined by thin layer chromatography (TLC) analysis on a silica gel 60 F254 plate (Merck, Darmstadt, Germany). Chromatographic purification was carried on silica gel columns 60 (0.063–0.200 mm or 0.040–0.063 mm, Merck), basic silica gel. Commercial reagents and solvents were used without additional purification. Abbreviations are used as follows: CDCl3, deuterated chloroform; DMSO-d6, dimethyl sulfoxide-d6; EtOAc, ethyl acetate; DMF, N,N-dimethylformamide; MeOH, methanol; THF, tetrahydrofuran; EtOH, ethanol; DMSO, dimethyl sulfoxide; NMP, N-methylpyrrolidone. High resolution mass spectra were recorded on a Finnigan MAT 95S mass spectrometer.

### 3.2. Chemical Synthesis

#### 3.2.1. General Procedure for the Preparation of Acylated Amino Acids

*N*,*O*-Bis(trimethylsilyl)acetamide (BSA, 2.49 mL, 10.05 mmol) was added to a solution of L-phenylalanine (0.83 g, 5.02 mmol) and dichloromethane (8 mL), and the reaction mixture was stirred at room temperature for 16 h. In another 100 mL reaction flask, 1,1'-carbonyldiimidazole (1.7 g, 10.48 mmol) was added to a solution of pyrazinecarboxylic acid (1 g, 8.06 mmol) and dichloromethane (17 mL). After stirring at room temperature for 16 h, the reaction mixture was cooled in a −40 °C bath and the above solution of L-phenylalanine-BSA was added dropwise over 30 min. The reaction mixture was stirred at room temperature for another 16 h, citric acid_(aq)_ (0.78 M, 17 mL) was added, and extracted with dichloromethane (10 mL × 3). The organic layers were separated, combined, dried over anhydrous Na_2_SO_4_ and concentrated. The crude product was recrystallized in dichloromethane/hexanes to give *N*-(2-pyrazinylcarbonyl)-L-phenylalanine (**8**, R = benzyl; R' = pyrazinyl, 0.96 g, 3.54 mmol, 70%) as a colorless solid. [11] Mp 142–146 °C, ^1^H-NMR (CDCl_3_) δ 9.34 (s, 1H), 8.73 (s, 1H), 8.51 (s, 1H), 8.18 (d, 1H), 7.28–7.20 (m, 5H), 5.12–5.05 (m, 1H), 3.37–3.21 (m, 2H); ^13^C-NMR (DMSO-*d*_6_) δ 172.4, 162.6, 147.8, 144.1, 143.5, 143.4, 137.4, 129.1, 128.2, 126.5, 53.5, 36.3.

#### 3.2.2. General Procedure for the Preparation of Dipeptide Boronates

*N-[(1S)-1-[[[(1R)-1-[(3aS,4S,6S,7aR)-Hexahydro-3a,5,5-trimethyl-4,6-methano-1,3,2-benzodioxaborol-2-yl]-3-methylbutyl]-amino]carbonyl]-2-phenyl]-2-pyrazincarboxamide* (**1**). Diethylisopropylamine (0.2 mL, 0.15 g, 1.19 mmol) was added to the solution of *N*-(2-pyrazinylcarbonyl)-L-phenylalanine (75 mg, 0.26 mmol), (*R*)-BoroLeu-(+)-Pinanediol (100 mg, 0.28 mmol) and HBTU (110 mg, 0.29 mmol) in DMF (2 mL) at 0 °C. The reaction mixture was stirred at room temperature for 16 h, the water (30 mL) was added, and it was extracted with EtOAc (10 mL × 3). The organic layers were combined, washed with citric acid_(aq)_ (0.1 *N*, 30 mL), NaHCO_3(aq)_ (0.1 *N*, 30 mL), sat. NaCl_(aq)_ (30 mL), dried over Na_2_SO_4_, filtered and conventrated. The crude product was purified by column chromatography (SiO_2_, EtOAc/hexanes, 1:1; R*_f_* 0.40) to give **1** [11] (100 mg, 0.19 mmol, 73%) as a colorless solid. ^1^H-NMR (CDCl_3_) δ 9.31 (s, 1H), 8.71 (s, 1H), 8.49 (s, 1H), 8.35 (d, *J* = 8.4 Hz, 1H), 7.26–7.20 (m, 5H), 5.98 (m, 1H), 4.80–4.77 (m, 1H), 4.29–4.26 (d, *J* = 10.8 Hz, 1H), 3.17–3.08 (m, 3H), 2.33–2.25 (m, 1H), 2.18–2.12 (m, 1H), 2.03 (t, *J* = 5.4 Hz, 1H), 1.80–1.91 (m, 2H), 1.47–1.56 (m, 1H), 1.43 (s,3H). 1.35 (d, *J* = 10.7 Hz, 1H), 1.31–1.47 (m, 2H), 1.28 (s, 3H), 0.86 (s, 3H), 0.85 (d, *J* = 5.0 Hz, 3H), 0.84 (d, *J* = 5.0 Hz, 3H); ^13^C-NMR (CDCl_3_) δ 170.5, 162.5, 147.1, 144.0, 142.5, 136.3, 129.2, 128.2, 126.6, 85.3, 77.5, 53.7, 51.3, 39.8, 39.4, 38.5, 38.3, 37.9, 35.7, 35.4, 28.4, 26.9, 26.1, 25.9, 23.8, 22.7, 21.8.

#### 3.2.3. General Procedure for the Hydrolysis of Boronic Esters

*(S)-(3-(3-Phenyl-2-(pyrazine-2-carboxamido)propanamido)propyl)boronic acid* (**13**). Potassium hydrogen difluoride (4.5 M, 0.41 mmol) was added to a solution of (*S*)-*N*-(1-oxo-3-phenyl-1-((3-(4,4,5,5-tetramethyl-1,3,2-dioxaborolan-2-yl)propyl)amino)propan-2-yl)pyrazine-2-carboxamide (19 mg, 0.043 mmol) and methanol (0.5 mL). The reaction mixture was stirred at room temperature for 4 h and concentrated. The residue was added with water (0.4 mL) and silica gel (8 mg, 0.13 mmol), and the suspension was stirred for another 5 h at room temperature. The reaction mixture was diluted with EtOAc (5 mL), filtered, dried over Na_2_SO_4_, and concentrated to give product **13** (10 mg, 0.028 mmol, 21%). ^1^H-NMR (acetone-d_6_) δ 9.18 (s, 1H), 8.82 (s, 1H), 8.65 (s, 1H), 8.47 (s, 1H), 7.50 (s, 1H), 7.28–7.15 (m, 5H), 4.90–4.83 (m, 1H), 3.24–3.13 (m, 4H), 1.55 (t, *J* = 7.2 Hz, 2H), 0.68 (t, *J* = 7.8 Hz, 2H); ^13^C-NMR (acetone-d_6_) δ 170.4, 162.3, 147.7, 144.5, 143.7, 143.2, 137.3, 129.4, 128.2, 126.5, 54.3, 41.3, 38.5, 29.6, 24.4; HRMS (ESI) [M−H]^−^ calcd for C_17_H_20_BN_4_O_4_ 355.1578, found 355.1575.

*(S)-Methyl 4-methyl-2-((S)-3-phenyl-2-(pyrazine-2-carboxamido)propanamido)pentanoate* (**11**): ^1^H-NMR (CDCl_3_) δ 9.31 (s, 1H), 8.71 (s, 1H), 8.50 (s, 1H), 8.32 (d, *J* = 8.4 Hz, 1H), 7.28–7.15 (m, 5H), 6.21 (d, *J* = 7.2 Hz, 1H), 4.81 (dt, *J* = 7.2 Hz, *J* = 7.2 Hz, 1H), 4.49, (m, 1H), 3.66 (s, 3H), 3.17, (d, *J* = 7.2 Hz, 2H), 1.54–1.42 (m, 3H), 0.82 (d, *J* = 5.5 Hz, 6H); ^13^C-NMR (CDCl_3_) δ 172.3, 170.2, 162.9, 147.4, 144.2, 144.1, 142.7, 136.2, 129.3, 128.5, 126.9, 54.4, 52.2, 50.9, 41.2, 38.4, 24.7, 22.5, 21.8 [11].

*N-((S)-1-(((S)-1-Hydroxy-4-methylpentan-2-yl)amino)-1-oxo-3-phenylpropan-2-yl)pyrazine-2-carboxamide* (**12**): ^1^H-NMR (CDCl_3_) δ 9.29 (d, *J* = 1.5 Hz, 1H), 8.71 (d, *J* = 2.4 Hz, 1H), 8.50 (d, *J* = 2.4 Hz, 1H), 8.42 (d, *J* = 8.1 Hz, 1H), 7.28–7.15 (m, 5H), 6.10 (br, 1H), 4.83 (m, 1H), 3.88 (m, 1H), 3.91-3.31 (m, 2H), 3.24–3.06 (m, 2H), 2.30–2.00 (br, 2H), 1.45–1.38 (m, 1H), 1.41 (dd, *J* = 6.3 Hz, *J* = 6.3 Hz, 2H), 0.75 (dd, *J* = 6.3 Hz, *J* = 1.9 Hz, 6H); ^13^C-NMR (CDCl_3_) δ 170.4, 163.0, 147.5, 144.2, 143.9, 142.8, 136.5, 129.4, 128.7, 127.1, 65.1, 55.0, 50.0, 39.7, 38.8, 24.6, 22.8, 22.1; HRMS (ESI) [M−H]^−^ calcd for C_20_H_25_N_4_O_3_ 369.1927, found 369.1922.

*(S)-N-(1-Oxo-3-phenyl-1-((3-(4,4,5,5-tetramethyl-1,3,2-dioxaborolan-2-yl)propyl)amino)propan-2-yl)pyrazine-2-carboxamide* (**14**): ^1^H-NMR (CDCl_3_) δ 9.30 (s, 1H), 8.70 (s, 1H), 8.51 (s, 1H), 8.41 (m, 1H), 7.28–7.18 (m, 5H), 4.76–4.70 (m, 1H), 3.22–3.07 (m, 4H), 1.50–1.43 (m, 2H), 1.19 (s, 12H), 0.84–0.77 (m, 2H); ^13^C-NMR (CDCl_3_) δ 169.9, 162.7, 147.3, 144.0, 142.7, 136.5, 129.2, 128.6, 126.9, 83.1, 54.9, 41.5, 38.8, 29.6, 24.7, 23.4, 14.1; HRMS (ESI) [M+Na]^+^ calcd for C_23_H_31_BN_4_O_4_Na 461.2336, found 461.2331.

*N-(2-(((R)-3-methyl-1-((3aR,4R,6R,7aS)-5,5,7a-trimethylhexahydro-4,6-methanobenzo[d][1,3,2]dioxaborol-2-yl)butyl)amino)-2-oxoethyl)pyrazine-2-carboxamide* (**15**): ^1^H-NMR (CDCl_3_) δ 9.33 (s, 1H), 8.68 (s, 1H), 8.49 (s, 1H), 7.85 (br, s, 1H), 4.33 (d, 2H), 3.98–3.92 (m, 2H), 2.82–2.92 (m, 1H), 2.43–2.35 (m, 1H), 2.28–2.22 (m, 1H), 2.13 (t, *J* = 5.4 Hz, 1H), 1.91–1.80 (m, 2H), 1.56–1.47 (m, 1H), 1.43 (s,3H). 1.35 (d, *J* = 10.7 Hz, 1H), 1.47–1.31 (m, 2H), 1.28 (s, 3H), 0.86 (s, 3H), 0.85 (d, *J* = 5.0 Hz, 3H), 0.84 (d, *J* = 5.0 Hz, 3H); ^13^C-NMR (CDCl_3_) δ 171.0, 162.7, 147.0, 144.3, 142.6, 86.2, 78.0, 53.9, 51.2, 40.5, 40.1, 39.5, 38.9, 38.4, 35.7, 29.2, 27.8, 27.0, 25.6, 24.4, 23.3, 22.3. 

*N-((S)-1-(((R)-3-Methyl-1-((3aR,4R,6R,7aS)-5,5,7a-trimethylhexahydro-4,6-methanobenzo[d][1,3,2]dioxaborol-2-yl)butyl)amino)-1-oxo-3-phenylpropan-2-yl)benzamide* (**16**): ^1^H-NMR (CDCl_3_) δ 7.71–7.70 (d, 2H), 7.48–7.45 (d, 2H), 7.26–7.20 (m, 6H), 4.84–4.75 (m, 1H), 4.30–4.27 (d, *J* = 6.9 Hz, 1H), 3.17–3.08 (m, 3H), 2.33–2.25 (m, 1H), 2.18–2.12 (m, 1H), 2.03 (t, *J* = 5.4 Hz, 1H), 1.91–1.80 (m, 2H), 1.56–1.47 (m, 1H), 1.43 (s,3H). 1.35 (d, *J* = 10.7 Hz, 1H), 1.47–1.31 (m, 2H), 1.28 (s, 3H), 0.86 (s, 3H), 0.85 (d, *J* = 5.0 Hz, 3H), 0.84 (d, *J* = 5.0 Hz, 3H); ^13^C-NMR (CDCl_3_) δ 170.4, 162.8, 137.2, 133.9, 131.6, 129.4, 128.5, 128.4, 127.1, 126.7, 55.2, 37.5, HRMS (FAB) [M+H]^+^ calcd for C_31_H_42_O_4_N_2_B 517.3238, found 517.3232.

*(S)-2-Acetamido-N-((R)-3-methyl-1-((3aR,4R,6R,7aS)-5,5,7a-trimethylhexahydro-4,6-methanobenzo[d][1,3,2]dioxaborol-2-yl)butyl)-3-phenylpropanamide* (**17**): ^1^H-NMR (CDCl_3_) δ 7.24–7.16 (m, 5H), 4.58–4.63 (m, 1H), 4.21–4.20 (d, *J* = 5.1 Hz, 1H), 3.05–2.98 (m, 3H), 2.33–2.25 (m, 1H), 2.18–2.12 (m, 1H), 2.03 (t, *J* = 5.4 Hz, 1H), 1.97 (s,3H) 1.80–1.91 (m, 2H), 1.47–1.56 (m, 1H), 1.43 (s,3H). 1.35 (d, *J* = 10.7 Hz, 1H), 1.31–1.47 (m, 2H), 1.28 (s, 3H), 0.86 (s, 3H), 0.85 (d, *J* = 5.0 Hz, 3H), 0.84 (d, *J* = 5.0 Hz, 3H); ^13^C-NMR (CDCl_3_) δ 171.3, 169.9, 136.6, 129.3, 128.4, 126.7, 85.5, 77.6, 53.8, 51.3, 39.9, 39.5, 38.5, 38.4, 38.0, 35.5, 28.5, 27.0, 26.2, 25.2, 23.9, 22.9, 21.9; HRMS (ESI) [M+Na]^+^ calcd for C_26_H_39_BN_2_O_4_Na 477.2901, found 477.2892.

*(R)-(3-Methyl-1-(2-(pyrazine-2-carboxamido)acetamido)butyl)boronic acid* (**18**): ^1^H-NMR (acetone-d_6_) δ 9.28 (s, 1H), 8.88 (s, 1H), 8.67 (s, 1H), 5.80–5.60 (m, 2H), 2.60–2.53 (m, 1H), 1.89 (s,3H), 1.65–1.51 (m, 1H), 1.40–1.12 (m, 2H), 0.88–0.77 (m, 6H); ^13^C-NMR (CDCl_3_) δ 171.0, 162.7, 147.0, 144.3, 142.6, 142.1, 77.4, 50.3, 29.6, 27.3, 27.0. 

*((R)-1-((S)-2-Benzamido-3-phenylpropanamido)-3-methylbutyl)boronic Acid* (**19**): ^1^H-NMR (acetone-d_6_) δ 7.85–7.81 (d, 2H), 7.48–7.45 (d, 2H), 7.26–7.20 (m, 6H), 4.89–4.79 (m, 1H), 3.31–3.21 (m, 2H), 3.14–3.11 (m, 1H), 1.68–1.55 (m, 1H), 1.40–1.12 (m, 2H), 0.88–0.77 (m, 6H); ^13^C-NMR (CDCl_3_) δ 171.0, 162.7, 141.2, 134.7, 133.4, 132.6, 77.4, 56.5, 30.7, 28.0, 27.8; HRMS (ESI) [M+Na]^+^ calcd for C_21_H_27_BN_2_O_4_Na 405.1962, found 405.1965.

*((R)-1-((S)-2-Acetamido-3-phenylpropanamido)-3-methylbutyl)boronic Acid* (**20**): ^1^H-NMR (acetone-d_6_) δ 7.29–7.23 (m, 5H), 4.95–4.87 (m, 1H), 3.12–3.09 (d, *J* = 7.8 Hz, 2H), 2.60–2.53 (m, 1H), 1.89 (s, 3H), 1.65–1.51 (m, 1H), 1.40–1.12 (m, 2H), 0.88–0.77 (m, 6H); ^13^C-NMR (CDCl_3_) δ 171.3, 169.9, 136.6, 129.3, 128.4, 126.7, 53.8, 51.3, 29.6, 27.9, 27.3, 27.0.

### 3.3. Biological Assay

#### 3.3.1. Cell Culture

The Sk-Hep1 cell line was obtained from American Type Culture Collection (Manassas, VA, USA). Cells were maintained in Dulbecco’s modified Eagle’s medium supplemented with 10% fetal bovine serum, 100 units/mL, penicillin G, 100 mg/mL streptomycin sulfate and 25 mg/mL amphotericin B in a 37 °C humidified incubator and an atmosphere of 5% CO_2_ in air. 

#### 3.3.2. Proteasome Activity Assay

A 20S Proteasome Activity Assay kit (Chemicon, Billerica, MA, USA) was used to determine the inhibition of proteasome in drug-treated Sk-Hep1 cells. Cell lysates were prepared, and the fluorogenic peptide substrate, Suc-Leu-Leu-Val-Tyr-AMC was used according to the manufacturer’s instructions. In brief, control or compound-treated cells were broken in a lysis buffer without protease inhibitors. Total cell lysate (50 μg) was incubated with 20 μmol/L of fluorogenic substrate Suc-Leu-Leu-Val-Tyr-AMC at 45 °C in 100 μL of assay buffer. Free AMC liberated by the substrate hydrolysis was quantified for 90 min at 1-min intervals on a microtiter plate fluorometrer (excitation, 355 nm; emission, 460 nm). The percentage of proteasome activity values (% control) were derived by dividing the slope obtained in the presence of bortezomib or compounds by the slope obtained in its absence ×100 [12].

#### 3.3.3. Western Blot

Lysates of Sk-hep1 treated with bortezomib and derivatives at the indicated concentrations for 24 h were prepared for immunoblotting of CIP2A, Akt (Akt1), p27, Cyclin E and PARP (Santa Cruz Biotechnology, Santa Cruz, CA, USA), p53, E2F-1, p-Akt (Ser473), caspase-8, caspase-9 and caspase-3 (Cell Signaling Technology, Danvers, MA, USA), β-Actin (Sigma, Steinheim, Germany).

#### 3.3.4. Cell Viability Analysis

The effect of bortezomib and its derivatives on Sk-hep1, Huh7, and Hep3B cell viability was assessed by the 3-(4,5-dimethylthiazol-2-yl)-2,5-diphenyltetrazolium bromide (MTT) assay in 12 replicates. 

#### 3.3.5. Apoptosis Analysis

Apoptotic cells were measured by flow cytometry (sub-G1). After treatment with compounds, cells were trypsinized, collected by centrifugation and resuspended in PBS. After centrifugation, the cells were washed in PBS and resuspended in potassium iodide (PI) staining solution. Specimens were incubated in the dark for 30 min at 37 °C and then analyzed with an EPICS Profile II flow cytometer (Coulter Corp., Hialeah, FL, USA). All experiments were performed in triplicate.

## 4. Conclusions

We synthesized a series of bortezomib derivatives that showed CIP2A inhibition activity, and the inhibition of CIP2A-Akt signaling pathway correlated with the cytotoxicity of these derivatives in HCC cells. Several compounds were found equally as potent as bortezomib in inhibition of CIP2A and cell growth. A mechanistic study showed that compound **1** also reduced expression of p-Akt after repressing the CIP2A signaling cascade. In addition, compound **1** provides a useful pharmacological tool to the study therapeutic relevance of HCC with unregulated CIP2A expression and drug resistance. These compounds will be tested in other cancers. Moreover, a series of new derivatives provide structure activity relationship which provide a direction for future lead optimization of CIP2A inhibitors. Therefore, these data suggest the potential value of anti-CIP2A agents in the treatment of cancers. Testing of compound **1** in an *in vivo* HCC model and pre-clincal studies are currently being pursued.

## Supplementary Material

Supplementary materials can be accessed at: http://www.mdpi.com/1420-3049/18/12/15398/s1.
