# Supplementary material for: Bortezomib Congeners Induce Apoptosis of Hepatocellular Carcinoma via CIP2A Inhibition
Source: Molecules. 2013 Dec 11;18(12):15398–411. doi: 10.3390/molecules181215398 (PMC6269665; doi:10.3390/molecules181215398)

Supplementary Material

Figure S1. Western blot analysis of CIP2A, Akt (Akt1), P-Akt (Ser473) and caspases 3 levels in Huh7 cells.

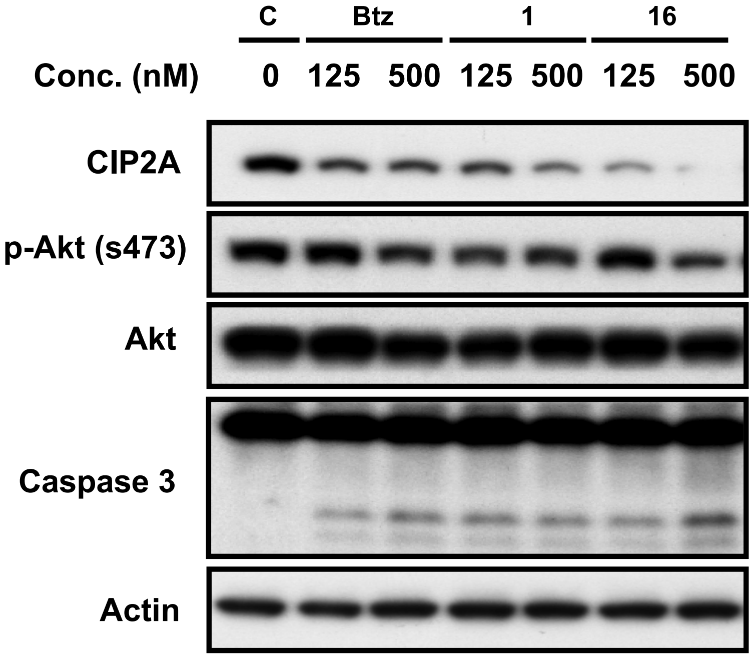

Figure S2. Effects of bortezomib and derivatives in Huh7 cell line. *Upper left*, for cell viability, cells were exposed to bortezomib and derivatives at 250 nM in DMEM with 5% FBS for 48 h. *Upper right*, for cell death ELISA, cell were exposed to bortezomib and derivatives at 500 nM in DMEM with 5% FBS for 24 h. Effects of Bortezomib and its derivatives in Hep3B cell line. *down left*, for cell viability, Cells were exposed to bortezomib and derivatives at 250 nM in DMEM with 5% FBS for 48 h. *down right*, Analysis of apoptotic cells of cells were done by flow cytometry (sub-G1) after cells were exposed to 500 nM bortezomib and derivatives for 24 h. * Difference is statistically significant ($p < 0.05$).

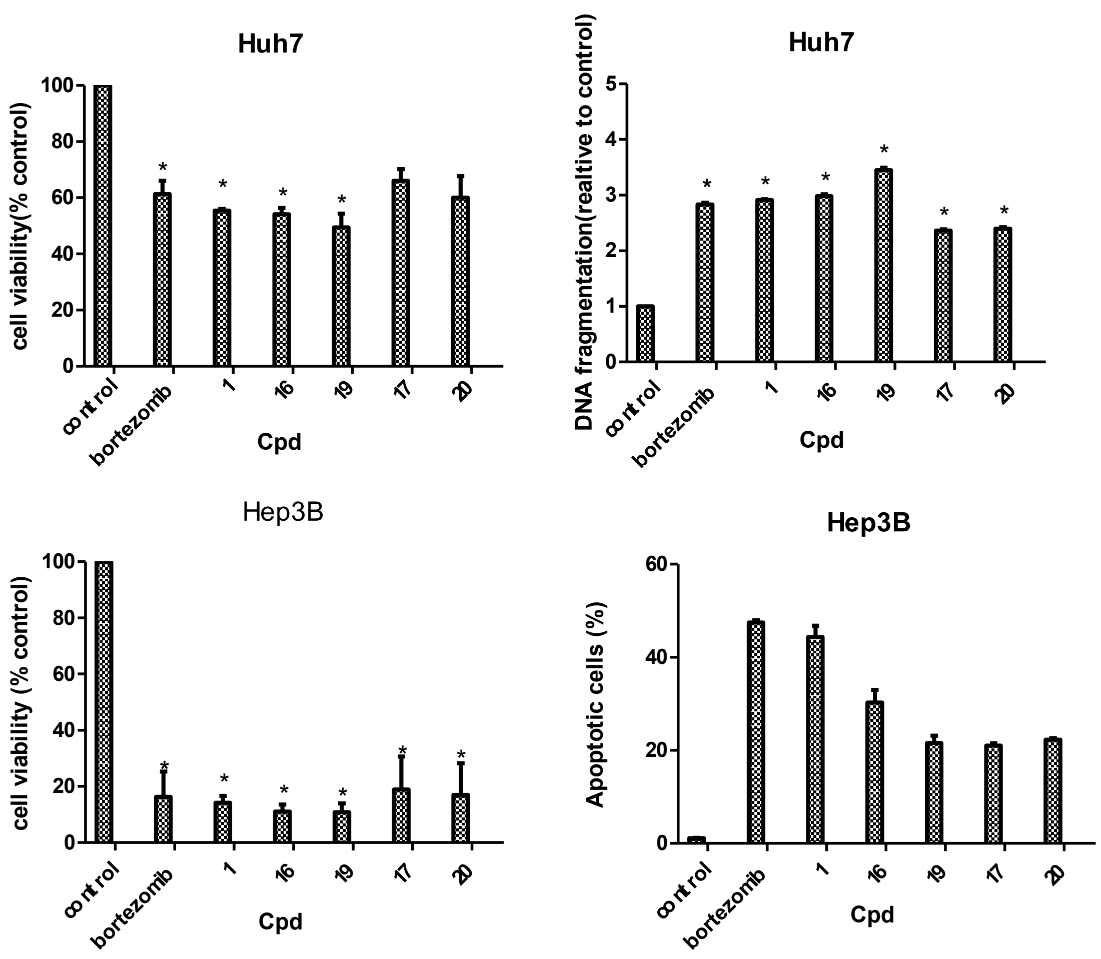

Supplement: Supplementary file 1 [file molecules-18-15398-s001.pdf]
